# Effect of hypoxemia on outcome in respiratory failure supported with extracorporeal membrane oxygenation: a cardinality matched cohort study

**DOI:** 10.1097/MAT.0000000000001835

**Published:** 2022-10-25

**Authors:** Alex Warren, Mikel A. McKie, Sofía S. Villar, Luigi Camporota, Alain Vuylsteke

**Affiliations:** 1 Division of Anaesthesia, Department of Medicine, University of Cambridge, Cambridge, UK; 2 Critical Care Unit, Royal Papworth Hospital NHS Foundation Trust, Cambridge, UK; 3 Biostatistics Unit, Cambridge Institute of Public Health, School of Clinical Medicine, University of Cambridge, Cambridge, UK; 4 Papworth Trials Unit Collaboration, Cambridge, UK; 5 Division of Asthma, Allergy and Lung Biology, King’s College London, London, UK; 6 Department of Critical Care, Guy’s & St. Thomas’s Hospitals, London, UK

**Keywords:** acute lung injury, acute respiratory distress syndrome, extracorporeal membrane oxygenation, extracorporeal life support, cardinality matching

## Abstract

Venovenous extracorporeal membrane oxygenation (ECMO) is recommended in adult patients with refractory acute respiratory failure (ARF), but there is limited evidence for its use in patients with less severe hypoxemia. Prior research has suggested a lower PaO_2_/FiO_2_ at cannulation is associated with a higher short-term mortality, but it is unclear whether this is due to less severe illness or a potential benefit of earlier ECMO support. In this exploratory cardinality-matched observational cohort study, we matched 668 patients who received venovenous ECMO as part of a national severe respiratory failure service into cohorts of ‘less severe’ and ‘very severe’ hypoxemia based on the median PaO_2_/FiO_2_ at ECMO institution of 68mmHg. Prior to matching, ICU mortality was 19% in the ‘less severe’ hypoxemia group and 28% in the ‘very severe’ hypoxemia group (RR for mortality = 0.69, 95% CI 0.54—0.88). After matching on key prognostic variables including underlying diagnosis, this difference remained statistically present but smaller: (23% vs. 30%, RR = 0.76, 95% CI 0.59—0.99). This may suggest the observed survival benefit of venovenous ECMO is not solely due to reduced disease severity. Further research is warranted to examine the potential role of ECMO in ARF patients with less severe hypoxemia.

## Introduction

Extracorporeal membrane oxygenation (ECMO) is recommended for organ support in eligible adults with severe acute respiratory failure (ARF) ^1^. A recent meta-analysis of the two randomized controlled trials of respiratory ECMO in adults showed a reduction in mortality at 60 days ^2^. In severe ARF, ventilator-induced lung injury is known to worsen outcome, with contributing factors that include high tidal volumes, ventilatory pressures and fraction of inspired oxygen (FiO_2_)^3–7^. It has been suggested that application of ECMO earlier in the course of severe ARF may improve outcome by reducing the time the lung is exposed to high mechanical ventilation settings and allowing a reduction in mechanical power, minimizing further lung injury ^8–10^.

In a subgroup analysis of the EOLIA trial, a randomized controlled trial (RCT) of ECMO in ARF, patients who were randomized at a PaO2/FiO2 of ≥ 66mmHg had a greater survival benefit from ECMO than those randomized at more severe hypoxemia (PaO_2_/FiO_2_ ratio < 66mmHg) ^10^. Further randomized trials powered to examine the hypothesis that initiation of ECMO in less severe ARF would lead to increased survival pose challenges and are unlikely to occur ^1, 11–13^. In the absence of RCT data, consensus opinion from expert groups recommends the use of causal inference methodology such as matching strategies on large observational datasets ^14^, particularly where confounding factors exist and are measured. In this case, factors such as the underlying diagnosis, pre-ECMO duration of mechanical ventilation and presence and severity of hypercapnia are likely to influence the relationship between severity of hypoxemia at ECMO institution and outcome.

The aim of this observational study was to explore the relationship between severity of hypoxemia at ECMO cannulation and short-term survival in matched cohorts of adult patients with severe ARF, correcting for potential confounding. This study hypothesized that after matching for confounders, patients who received ECMO at a higher PaO_2_/FiO_2_ ratio would have imporved short-term outcomes.

## Study Design and Methods

A registry study of the NHS ECMO service reported the short-term outcomes of 1,205 adults with refractory respiratory failure treated over a six-year period ^15^. The service consists of six specialist, high-volume ECMO centers who provide an ECMO service for patients with severe ARF from a reversible cause. Patients are accepted by the national service if they have failed conventional management of ARF, which includes the routine use of at least one trial of prone positioning unless contra-indicated. Referral criteria include a Murray lung injury score ^16^ of 3.0 or higher and/or uncompensated hypercapnia with arterial pH ≤7.20, though these are not mandated.

**Figure 1:**
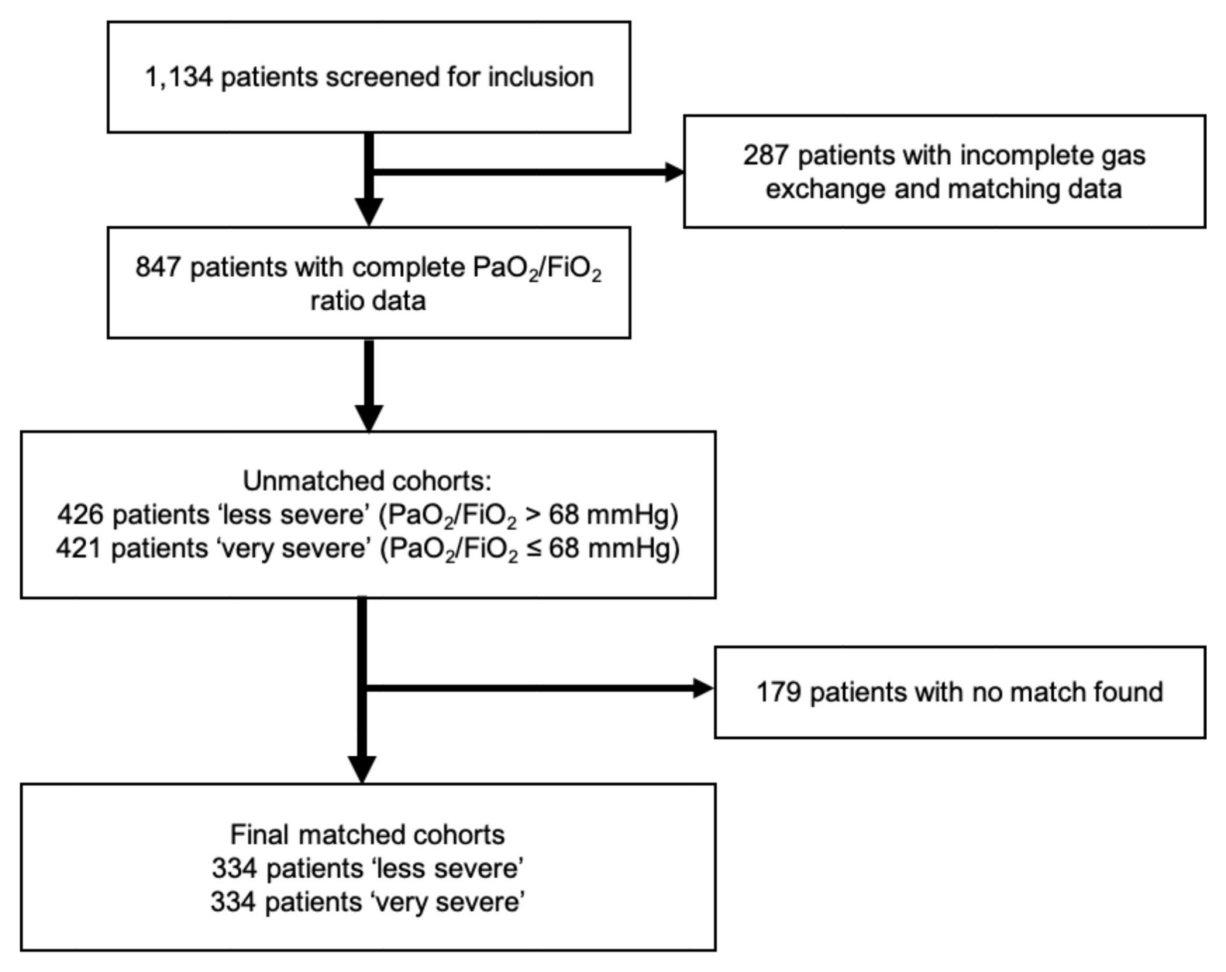
Flow diagram for a cardinality-matched cohort study of severity of hypoxaemia at ECMO institution.

The NHS ECMO registry was created by linking the Extracorporeal Life Support Organisation data entry form to the NHS England database for each patient treated by the national respiratory failure service ^15^. All patients from the registry were screened for inclusion in this pre-planned secondary analysis. Patients were excluded if the initial support modality was not veno-venous ECMO, as this suggests a high likelihood of other extra-pulmonary factors (e.g., shock) determining the timing of ECMO. A flow diagram for the study is given in Figure 1.

In addition to demographic data, biochemical data were extracted from the arterial blood gas closest to the decision to institute ECMO. Only cases with complete data for those variables used for matching were included, and there was no imputation of missing data. Cut-off for ‘very severe’ vs. ‘less severe’ hypoxemia was based on the median PaO_2_/FiO_2_ ratio of 68 mmHg calculated from the entire cohort prior to exclusion of cases with missing data for matching variables. While the use of any dichotomizing cut-off has statistical limitations, this was chosen to allow two similar-sized groups and enable the study design; the cut-off of 68 mmHg was also similar to that of 66 mmHg in the prospective EOLIA sub-analysis ^10^.

Cardinality matching was first described in 2014 and uses an iterative, exhaustive search procedure which compares all possible matched pairs for a given dataset, classification and confounding variables, and acceptable level of heterogeneity, and selects the subgroups with the greatest sample size (or ‘cardinality’) ^17^. Unlike traditional matching methods, it does not require the use a logistic regression-based propensity score. Prior studies have demonstrated a tendency for cardinality matching to return larger sample sizes while achieving an exact level of balance in smaller studies as compared to propensity-score based methods ^18^. A further advantage is the ability to set minimum acceptable differences on pre-specified variables. In this study, we used the methods described by Zubizaretta ^17, 19^ and set optimizer criteria of a maximum of 0.1 standardized mean difference (SMD) between groups for continuous variables and exact matches for categorical variables.

Variables were entered into the model based on clinical reasoning; variables with known association with outcome — in particular, age, primary diagnosis, PaCO_2_ and center — were forced into the model. PaO_2_ and arterial oxygen saturation (SaO_2_) were excluded from the model *a priori* as they were likely to be co-linear with the classification variable. Controls were matched to cases in a 1:1 ratio, with no re-use of controls. After matching was performed, SMD was re-calculated to assess variable balance. A traditional propensity-score-modelling with nearest-neighbor matching analysis was also performed, but resulted in lower sample size and inadequate balance on some variables (reported in Supplementary Appendix); cardinality matching was thus selected as the optimal method for the primary analysis.

The primary outcome, mortality at discharge from the ICU at the ECMO center (*ICU mortality*), was calculated between both unmatched and matched cohorts and compared with risk ratios and their 95% confidence intervals. For clarity, both parametric and nonparametric variables are presented as mean ± SD given the use of SMD for assessment of balance. Secondary outcomes of continuous variables were compared with Mann-Whitney U test. A pre-specified secondary analysis was conducted on the relationship between hypoxemia at ECMO initiation and other key potential confounders, including duration of mechanical ventilation prior to initiation of ECMO, pH and PaCO_2_.

All statistical analysis was performed using R 4.0.1 (R Core Team, Vienna, Austria), with packages “MatchIt” for PSM, “designmatch” in conjunction with the CPLEX optimiser (IBM, Armonk, NY, US) for cardinality matching, and “fmsb” for some statistical analyses. All code can be made available on request.

### Ethical approval and registration

This study used routinely collected anonymized data and thus the need for formal research ethics committee approval was waived under the terms of the NHS Health Research Authority. The study was registered with the National Clinical Trials registry (NCT03981393).

## Results

### Identification of study cohort

After exclusion of patients who received ECMO in an initial configuration other than veno-venous, 1,134 patients were screened for inclusion. A total of 847 patients had sufficient data on PaO_2_/FiO_2_ ratio at decision-to-cannulate and other key matching variables for inclusion. A flow diagram for the study is given in Figure 1. The median PaO_2_/FiO_2_ ratio within the 847 included patients was 68 mmHg (9.0 kPa), similar to the cut-off of 68 mmHg (9.1 kPa) derived from the entire cohort.

The characteristics for the total cohort are presented in Table 1. ICU mortality, the primary outcome, was 24% in the 847 included patients (n = 203), similar to the original study from which the cohorts are derived ^15^. In unmatched analysis, the ‘less severe hypoxemia’ group (PaO_2_/FiO_2_ ratio > 68 mmHg) contained 426 patients with mean PaO_2_/FiO_2_ ratio of 116 ± 97 mmHg (15.5 ± 12.9 kPa); the ‘very severe hypoxemia’ group contained 421 patients with mean PaO_2_/FiO_2_ ratio of 56 ± 8 mmHg (7.4 ± 1.1 kPa). Factors associated with cannulation at ‘less severe hypoxemia’, as defined by SMD > 0.1, included: lower age (mean 42.5 vs. 44.8 years), primary diagnosis of asthma (18% vs. 2%), admission to center 5 (34% vs. 29%), lower PEEP (mean 10 vs. 13 cmH_2_O), lower pH (mean 7.17 vs. 7.19), higher PaCO_2_ (mean 80 vs. 65 mmHg / 10.7 vs. 8.7 kPa), and longer duration of pre-ECMO mechanical ventilation (mean 91 vs. 78 hours).

Prior to cardinality matching, decision to cannulation for ECMO at a higher PaO_2_/FiO_2_ ratio was associated with a significant reduction in ICU mortality (19% (82/421) in the ‘less severe hypoxemia group’ vs. 28% (121/426) in the ‘very severe hypoxemia’ group, risk ratio for mortality in less severe hypoxemia group = 0.69, 95% confidence interval = 0.54—0.88).

### Selection of matching strategies

**Figure 2:**
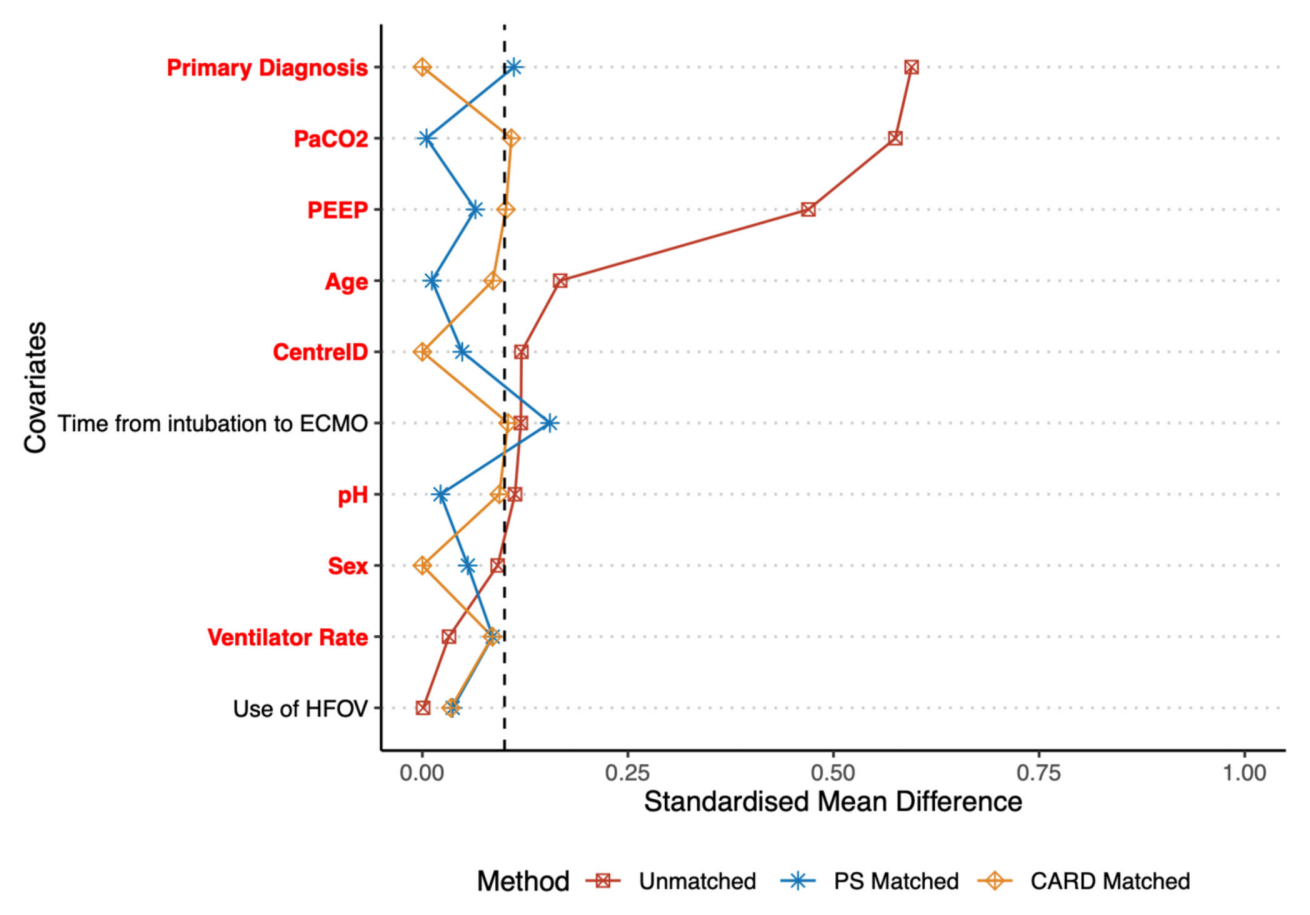
Standardised mean difference of variables in unmatched and matched cohorts of patients receiving ECMO at PaO_2_/FiO_2_ ≤ 68 mmHg vs. PaO_2_/FiO_2_ > 68 mmHg. ECMO = extracorporeal membrane oxygenation; PEEP = positive end-expiratory pressure; HFOV = high-frequency oscillatory ventilation; PS = propensity-score; CARD = cardinality matching. Variables where balance was improved by matching strategy are displayed in red.

Cardinality matching produced two cohorts of 334 patients each (total sample size n = 668). Balance was acceptable (in terms of SMD < 0.1) on all variables except duration of pre-ECMO mechanical ventilation, (SMD = 0.10), PEEP (SMD = 0.10), and PaCO_2_ (SMD = 0.11), all of which were negligibly above the threshold. In particular, balance was excellent (SMD = 0.00) on the key matching variable of primary diagnosis, ensuring that all included patients were matched with a patient with an identical underlying aetiology of respiratory failure. Further details on assessment of the matching strategy are given in the Supplemntary Appendix. Covariate balance pre-and post-matching is shown in Figure 2. The final cohorts used for matched analysis are shown in Table 2.

### Relationship between severity of hypoxemia and outcome

After cardinality matching, the ICU mortality rate in patients who received ECMO with ‘less severe hypoxemia’ was 23% (76/334) compared to 30% (99/334) in the ‘very severe hypoxemia’ group. The estimated relative risk for ICU mortality in the ‘less severe hypoxemia’ group was 0.76 (95% confidence interval = 0.59—0.99).

There was no significant between-group difference with regards to ICU length of stay at the ECMO center (‘less severe hypoxemia’ group median = 16.7 days, IQR 10.8—26.9; ‘very severe hypoxemia’ group = 18.0 days, IQR 12.2—28.0, p = 0.34) or duration of ECMO support (‘less severe hypoxemia’ group median 8.6 days, IQR 5.0—16.9; ‘very severe hypoxemia’ group = 9.6 days, IQR 5.5—16.0).

### Role of PaCO_2_, duration of mechanical ventilation, and underlying primary diagnosis

**Figure 3:**
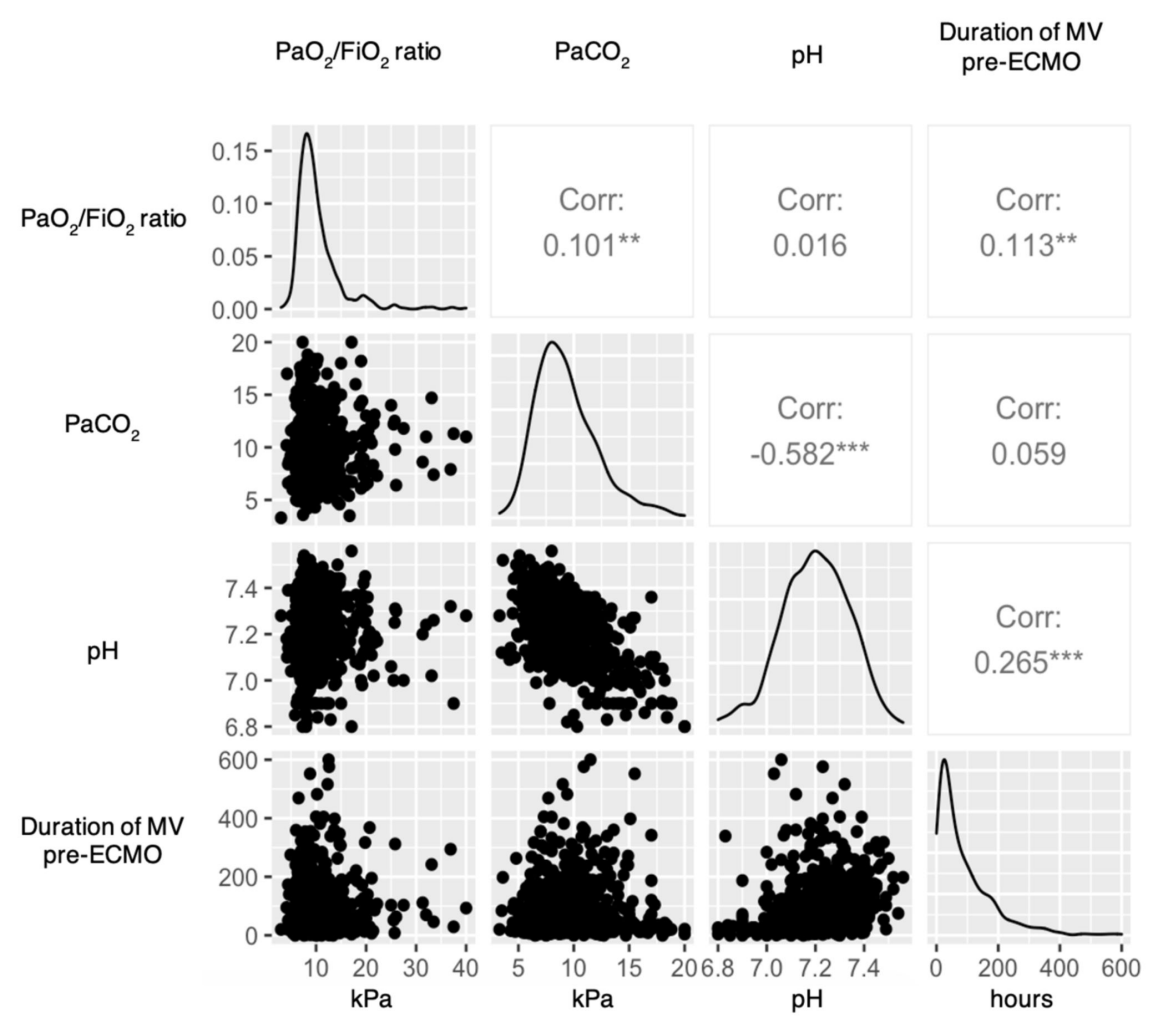
Distribution and correlation of pre-ECMO variables in two matched cohorts of patients receiving ECMO for acute respiratory failure (total n = 668) ECMO = extracorporeal membrane oxygenation; MV = mechanical ventilation; Corr = Pearson’s correlation coefficient. ** = p < 0.01; *** = p <0.001

Correlations between PaO_2_/FiO_2_ ratio, PaCO_2_, pH and duration of pre-ECMO ventilation and distributions of these variables in the combined matched cohorts are given in Figure 3; only PaCO_2_ and pH demonstrated a strong positive correlation (r = –0.57, p < 0.001). Post matching, median duration of pre-ECMO ventilation in the ‘less severe hypoxemia’ cohort was 67.5 (IQR 25—127) hours but 48.0 (IQR 22— 122) hours in the ‘very severe hypoxemia’ cohort (p = 0.028).

There was no relationship observed between PaCO_2_ and survival in either the total cohort prior to matching or the matched cohorts; shorter duration of pre-ECMO mechanical ventilation was associated with increased survival in the pre-matching cohort, but not in either of the matched cohorts (Table 2).

Following the observation that of 85 patients with an underlying diagnosis of asthma included in the screening cohort, only 18 were included in matching, a brief secondary analysis of this subgroup of patients with asthma was undertaken. Compared to patients with other underlying diagnoses, patients with asthma had significantly higher median PaCO_2_ (102 mmHg, IQR 79—122 (13.7 kPa, IQR 10.5—16.3) vs. 64 mmHg, IQR 53—81 (8.6 kPa, IQR 7.1— 10.8), p <0.001), significantly shorter duration of pre-ECMO mechanical ventilation (32 (IQR 16—82) vs. 53 (IQR 23—120) hours, p = 0.004), and were more likely to survive (94% (80/85) vs. 74% (564/762), p < 0.001).

## Discussion

In this cardinality-matched cohort study, ARF patients who received ECMO at a PaO_2_/FiO_2_ ratio greater than 68 mmHg (9.1 kPa) had a significantly lower mortality even after adjustment for potential measured confounders. The difference in mortality was smaller following cardinality matching (23% vs 30%, RR 0.76) compared to the unmatched analysis (19% vs 28%, RR 0.69), suggesting the presence of mediating factors; this is likely to represent the effect of ventilatory parameters (e.g. ability to provide ‘lung protective’ ventilation and reduce mechanical power delivered to the lung) which we were unable to measure. Included patients had a wide variety of underlying aetiologies, but this was matched completely across both cohorts, suggesting the effect is not restricted to a single aetiology of respiratory failure. While the matching strategy was well-balanced with regard to variables known to impact mortality in ARF patients receiving ECMO (e.g. age, underlying diagnosis, PaCO_2_, and duration of pre-ECMO mechanical ventilation), it did exclude the majority of asthmatic patients, identifying them as a distinct clinical group.

### Relationship between timing of ECMO and mortality in ARF

In the past, ECMO support was reserved for patients with only the most severe ARF. In the EOLIA trial, however, patients randomized at PaO_2_/FiO_2_ ratio of ≥ 66mmHg / 8.8 kPa had a relative risk for 60-day mortality of 0.56, compared to 1.04 in those randomized at PaO_2_/FiO_2_ ratio < 66mmHg / 8.8kPa ^10^; similarly, our observational study suggests that within a well-established, centralized system for ECMO provision in respiratory failure, patients with less severe hypoxemia had better short-term outcomes than those with severe hypoxemia, even when correcting for PaCO_2_ and known prognostic factors. Despite the use of cardinality matching, it is likely that some unmeasured confounding also exists.

There are a number of potential explanations for these findings. Firstly, it is possible that the observed association between ECMO provision at ‘less severe’ hypoxemia and outcome may not be causal, i.e. that there are unknown or unmeasured confounders underlying the observed association. Patients who present *in extremis* with rapidly deteriorating gas exchange — the so-called ‘crash and burn’ patient — are likely to both receive ECMO at lower PaO_2_/FiO_2_ ratios and have worse outcomes simply by virtue of their disease severity which may include extra-pulmonary organ failures. While the cardinality-matched analysis was balanced ‘acceptably’ on the variable of pre-ECMO duration of ventilation using parametric methods, there was a difference in the between-group medians. This may represent a slightly higher proportion of ‘crash- and-burn’ patients in the ‘very severe hypoxemia’ group. However, there was no correlation observed between PaO_2_/FiO_2_ ratio and pre-ECMO duration of ventilation in either the matched cohorts or the cohort as a whole, suggesting that this did not greatly affect the overall outcome.

Alternatively, observational and experimental data have demonstrated that ECMO allows for a significant reduction in mechanical ventilator power delivered to the alveoli — in one large study by up to 75% ^9^ — as well as significantly reducing biomarkers of lung injury ^20^. Patients with more severe disease are likely to have greater susceptibility to ventilator-induced lung injury due to inhomogeneity and smaller volume of healthy lung available for gas exchange ^21^. It is possible that establishment of ECMO in ‘less severe’ hypoxemia — as ‘lung rest’ rather than ‘rescue therapy’ — may reduce the duration of injurious ventilation and thus improve outcome. The findings of this present study would support this hypothesis, however this patient group (in this study consisting of a cohort with mean PaO_2_/FiO_2_ ratio of 105 ± 77 mmHg) has not been as well studied in terms of the potential benefits of ECMO compared to those with ‘refractory’ respiratory failure. We would stress that these theoretical benefits must be balanced against the substantial risks, complications and resource implications of ECMO, and require validation in prospective, randomized trials.

The relationship between disease severity as measured by hypoxemia and duration of pre-ECMO ventilation is complex. In this study, patients with ‘very severe’ hypoxemia had a shorter duration of pre-ECMO ventilation than the ‘less severe’ group. This suggests that, despite the classical Berlin definition of a progression through ‘mild’, ‘moderate’ and ‘severe’ ARF ^22^, severity is often maximal at presentation to critical care. While other studies have demonstrated an association between increased duration of pre-ECMO mechanical ventilation and decreased survival ^23, 24^, it is notable that when cohorts were matched on oxygenation in this study, this no longer appeared to affect mortality. It is clear that patients with prolonged ventilation and severe disease have very poor outcomes, as in the crossover group of EOLIA ^10^.

Some patients receive ECMO for refractory hypercapnia despite adequate oxygenation; however, the median PaCO_2_ and pH were identical between the matched cohorts, with no correlation observed between these indices and PaO_2_/FiO_2_ ratio. Patients with asthma, who are known to have very high survival rates ^15^, were far more likely to require ECMO due to hypercapnia. Most were excluded by the matching strategy, and the proportion of patients with asthma are identical between the cohorts, so this will not have biased the main analysis. Incidentally, this an advantage of the cardinality matching methodology employed, which identified a distinct phenotype that cannot be adequately matched with a control group, in this case asthma patients physiologically distinct from those with classical ‘acute respiratory distress syndrome’ as a cause of ARF.

### Learning from observational data and use of cardinality matching

The ideal study design to answer our research question — whether institution of ECMO in ARF patients with less severe hypoxemia improves outcome — would be a randomized controlled trial. Even with advanced observational methodology such as cardinality matching, randomization remains the ideal way to eliminate unobserved confounding in causal inference. An adequately powered RCT to evaluate earlier institution of ECMO based on the estimated difference in mortality in this study (23% vs 30%) would require over 600 patients per arm to detect a meaningful absolute mortality reduction of 7%. Given that the two contemporary ECMO RCTs have recruited nowhere near this number, and that ECMO trials have historically under-recruited ^13^, randomized trials of this size are unlikely to happen.

Given these limitations, critical care researchers are increasingly trying to answer such questions with observational data using ‘causal inference’ based methodology such as matched cohort studies ^14^. Unlike traditional propensity-score-based methods, cardinality matching has the advantage of not requiring logistic regression modelling or propensity score estimation, and may increase statistical power and balance, as it did on our dataset ^25^. Despite significant advantages, it is not yet used frequently in clinical medicine and this is one of the first ever examples of its application. We would suggest that given the unfeasibility of RCTs in some research questions, particularly regarding complex therapies such as ECMO, use of large, well curated observational datasets with methodologies such as cardinality matching may be a useful tool to gain further insights.

Results from cardinality-matched studies could also influence the design of adaptive trials such as response adaptive randomization (RAR), which was attempted over 35 years ago in a trial of neonatal ECMO ^26^. In this case between-group imbalance led to controversy and a second fixed design RCT ^27^. In the future, cardinality-matched techniques could be used to influence future adaptive trials in order to maximize patient response whilst balancing trial power and inferences.

### Limitations of this study

As stated above, our retrospective, exploratory study has multiple limitations. Our data are observational, and we cannot exclude unmeasured confounding. The UK National Health Service (NHS) ECMO registry collected only the mortality at discharge from the ICU at the ECMO center, so we were limited to this rather than a more long-term or patient-centered outcome. We excluded a proportion of cases due to missing data, which may induce bias, however the median PaO_2_/FiO_2_ of 68 mmHg (9.0 kPa) was effectively identical to the median of 68 mmHg (9.1 kPa) in the cohort prior to their removal, suggesting the included cohort remains robust and representative.

A significant limitation of this study is that only single time-points of gas exchange data, PaO_2_/FiO_2_, PEEP were available for analysis; and, crucially, no data were available on lung-protective ventilation settings such as driving pressure (ΔP), plateau pressure and tidal volume which are likely to have influenced outcome by varying the risk of ventilatory induced lung injury ^7^. In clinical practice, the rate of deterioration of gas exchange parameters and the response, or lack thereof, to other therapies such as prone positioning or recruitment maneuvers, will also influence the decision to refer for ECMO support, rather than a single PaO_2_/FiO_2_ measurement. In ARF, the PaO_2_/FiO_2_ ratio is not static but varies with mean airway pressure, FiO_2_ and hemodynamics ^28, 29^. Unfortunately, this was a feature of the registry from which the data was derived and could not be collected retrospectively.

## Interpretation

In this exploratory, retrospective study, patients with ARF who received ECMO at PaO_2_/FiO_2_ ratio > 68 mmHg / 9.1 kPa had improved short-term outcomes compared to cardinality-matched controls with PaO2/FiO2 ≤ 68mmHg / 9.1 kPa but identical PaCO2, duration of pre-ECMO ventilation and underlying diagnoses. While this may be due to the limitations of our study, including use of single time-points and lack of mechanical ventilation parameters, these findings support the hypothesis that patients with less severe hypoxemia who cannot be ventilated in a ‘lung-protective’ fashion may derive greater benefit from ECMO than those with refractory respiratory failure. Further research is required to explore the potential benefits of ECMO in ARF patients with less severe hypoxemia.

## Supplementary Material

Supplementary File

## Figures and Tables

**Table 1:** Characteristics of 847 patients identified as receiving veno-venous ECMO in the NHS ECMO registry prior to matching

	All patients (n = 847)	Less severe hypoxia n = 426	Very severe hypoxia n = 421	SMD
Age (years)	43.6 ± 14	42.5 ± 14	44.8 ± 13	0.17
Male sex	476 (56%)	230 (55%)	246 (58%)	0.09
Primary diagnosis§				0.59
Aspiration pneumonitis	35 (4%)	13 (3%)	22 (5%)	
Asthma	85 (10%)	76 (18%)	9 (2%)	
Bacterial pneumonia	167 (20%)	77 (18%)	90 (21%)	
Burns	9 (1%)	4 (1%)	5 (1%)	
Trauma	26 (3%)	11 (3%)	15 (4%)	
Viral pneumonia	194 (23%)	88 (21%)	106 (25%)	
Other (respiratory)	133 (16%)	62 (15%)	71 (17%)	
Other (non-respiratory)	117 (14%)	46 (11%)	71 (17%)	
				0.12
Centre				
1	167 (20%)	80 (19%)	87 (21%)	
2	116 (14%)	57 (13%)	59 (14%)	
3	151(18%)	70 (16%)	81 (19%)	
4	21 (2%)	9 (2%)	12 (3%)	
5	266 (31%)	143 (34%)	123 (29%)	
6	126 (15%)	62 (15%)	64 (15%)	
PEEP (cmH_2_O)	12 ± 5.2	10 ± 5.3	13 ± 4.9	0.47
Ventilator rate (/min)	12 ± 4.1	12 ± 4.7	12 ± 3.5	0.03
Use of HFOV	6 (<1%)	3 (<1%)	3 (<1%)	0.00
SaO_2_	87 ± 9.5	91 ± 8.0	83 ± 9.2	0.89
pH	7.19 ± 0.15	7.17 ± 0.15	7.19 ± 0.14	0.11
PaO_2_ (mmHg)	71 ± 43	89 ± 55	55 ± 8	0.87
FiO_2_	91 ± 15	83 ± 17	99 ± 9.0	1.25
PaO_2_/FiO_2_ ratio (mmHg)	86 ± 76	116 ± 98	56 ± 8	0.88
PaCO_2_ (mmHg)	73 ± 27	80 ± 31	65 ± 20	0.58
Duration of pre-ECMO ventilation (h)	84 ± 103	91 ± 102	78 ± 104	0.12

§ May not sum to 100% due to rounding/omitted categories.

ECMO = extracorporeal membrane oxygenation. SMD = standardized mean difference. PEEP = positive end-expiratory pressure. HFOV = high-flow oscillation ventilation. As SMD was used as matching criteria, continuous data are presented as mean ± SD.

**Table 2:** Cardinality-matched cohorts of patients receiving venovenous ECMO at less severe (PaO_2_/FiO_2_ > 66mmHg) and very severe (PaO_2_/FiO_2_ ≤ 66mmHg) hypoxia.

	Less severe hypoxia n =	Very severe hypoxia n =	SMD
Age (years)	44.4 ± 14	45.6 ± 13	0.09
Male sex	196 (59%)	196 (59%)	0.00
Primary diagnosis§			0.00
Aspiration pneumonitis	13 (4%)	13 (4%)	
Asthma	9 (3%)	9 (3%)	
Bacterial pneumonia	74 (22%)	74 (22%)	
Burns	4 (1%)	4 (1%)	
Trauma	11 (3%)	11 (3%)	
Viral pneumonia	86 (26%)	86 (26%)	
Other (respiratory)	58 (17%)	58 (17%)	
Other (non-respiratory)	45 (13%)	45 (13%)	
			0.00
Centre			
1	54 (16%)	54 (16%)	
2	49 (15%)	49 (15%)	
3	52 (16%)	52 (16%)	
4	8 (2%)	8 (2%)	
5	120 (36%)	120 (36%)	
6	51 (15%)	51 (15%)	
PEEP (cmH_2_O)	12 ± 4.5	12 ± 4.5	0.10
Ventilator rate (/min)	13 ± 4.7	12 ± 3.7	0.09
Use of HFOV	3 (1%)	2 (1%)	0.03
SaO_2_	91 ± 7.7	84 ± 7.4	0.89*
pH	7.21 ± 0.13	7.19 ± 0.14	0.09
PaO_2_ (mmHg)	85 ± 55	56 ± 8	0.76*
FiO_2_	85 ± 15	99 ± 4.1	1.21*
PaO_2_/FiO_2_ ratio (mmHg)	105 ± 77	56 ± 8	0.89*
PaCO_2_ (mmHg)	71 ± 22	69 ± 21	0.11
Duration of pre-ECMO ventilation (h)	97 ± 99	86 ± 113	0.10

§ May not sum to 100% due to rounding/omitted categories.

ECMO = extracorporeal membrane oxygenation. SMD = standardized mean difference. PEEP = positive end-expiratory pressure. HFOV = high-flow oscillation ventilation. As SMD was used as matching criteria, continuous data are presented as mean ± SD.
