# Supplementary material for: Effect of hypoxemia on outcome in respiratory failure supported with extracorporeal membrane oxygenation: a cardinality matched cohort study
Source: ASAIO J. Author manuscript; Available in PMC 2022 Dec 5. (PMC7613891; doi:10.1097/MAT.0000000000001835)
Supplement: Supplementary File [file EMS153898-supplement-Supplementary_File.pdf]

Supplementary Appendix:

1. Methodology of matching strategies

Matching was performed using two methods: traditional propensity score modelling with nearest-neighbor matching (PSM-NN); and cardinality matching. Cardinality matching was employed using the methods described by Zubizaretta and set optimizer criteria of a maximum of 0.1 standardized mean difference (SMD) between groups for continuous variables and exact matches for categorical variables. PSM-NN was conducted using an iterative, parsimonious model-building approach with a logistic regression model to build a model predicting group assignment, and nearest-neighbor matching with a maximum caliper of 0.1 standard deviations.

Variables were entered into the models based on clinical reasoning; variables with known association with outcome (age, primary diagnosis, $PaCO_2$, pH, center , duration of pre-ECMO mechanical ventilation, PEEP) — were forced into the model. PaO_2 and arterial oxygen saturation (SaO_2) were excluded from the model *a priori* as they were likely to be co-linear with the classification variable. Controls were matched to cases in a 1:1 ratio, with no re-use of controls.

After matching was performed, SMD was re-calculated to assess variable balance. The PSM-NN model was optimized by examination of co-variance between variables and introduction of any variables to improve balance. Both matching strategies were assessed for covariate balance (as defined by number of variables by <0.1 SMD between groups) and sample size, and the matching strategy with the greater power and balance chosen for analysis.

2. Comparison of matching strategies and selection of primary strategy

Cardinality matching produced two cohorts of 334 patients each (total sample size n = 668), while propensity-score-modelling with nearest-neighbor matching (PSM-NN) produced two smaller cohorts of 295 patients each (total sample size = 590). Balance was comparable with cardinality matching compared with PSM-NN (Figure 2). PSM-NN achieved acceptable balance (SMD < 0.10) on all but two included variables, duration of pre-ECMO mechanical

ventilation (SMD = 0.16) and primary diagnosis (SMD = 0.11). Cardinality matching achieved acceptable balance on all variables except duration of pre-ECMO mechanical ventilation, (SMD = 0.10), PEEP (SMD = 0.10), and $PaCO_2$ (SMD = 0.11). Each of these, however, was a significant improvement on the unmatched cohorts (main text Figure 2).

Given the comparable balance between covariates with cardinality matching with a substantial increase in sample size and therefore potentially increased statistical power, we considered cardinality matching as the superior methodology for the final study cohorts. The final cohorts used for matched analysis are given in Table 1 (main text).

Supplementary Table 1 – SMD for all variables in non-matched, PSM-NN and cardinality matched data.

covariates	Unmatched	PSM.matched	CARD.matched
Centre	0.12050493	0.04852562	0
Age	0.16763109	0.01161222	0.08575049
Duration of pre-ECMO mechanical ventilation	0.11980344	0.15520278	0.10425726
Sex	0.09159274	0.05530013	0
Primary Diagnosis	0.59494574	0.11126989	0
pH	0.11269917	0.02210979	0.09371302
FiO_2	1.24600382	1.07533977	1.21093938
SaO_2	0.8930665	0.78660297	0.88859714
PaO_2	0.86766718	0.83192493	0.762833
$PaCO_2$	0.57525297	0.00506168	0.10821966
PaO_2/FiO_2 ratio	0.88142464	0.71838846	0.88825225
PEEP	0.46952304	0.06449287	0.10162386
Ventilator rate	0.03238058	0.0855964	0.0851446
Use of HFOV	0.00099725	0.03698634	0.03474191
Age (days)	0.16899099	0.01379942	0.08745247
Transport	0.07410324	0.08532862	0.06205666
Repeat ECMO	0.01773227	0.02309401	0.0190123
ICULOS	0.03607887	0.0504895	0.042726
Mobile ECMO	0.06707207	0.03483439	0.070032
Duration of ECMO therapy	0.01455317	0.03063309	0.0429081
